# Seeking support for abortion online: a qualitative study of women’s experiences

**DOI:** 10.1136/bmjsrh-2023-202083

**Published:** 2024-02-09

**Authors:** Rachel Victoria Wilson-Lowe, Carrie Purcell, Ruth Lewis, Lisa McDaid

**Affiliations:** 1 Social and Public Health Sciences Unit, University of Glasgow, Glasgow, UK; 2 Faculty of Wellbeing, Education and Langauge Studies, The Open University, Milton Keynes, UK; 3 MRC/CSO Social and Public Health Sciences Unit, University of Glasgow, Glasgow, UK; 4 Institute for Social Science Research, The University of Queensland, Saint Lucia, Queensland, Australia

**Keywords:** abortion, induced, qualitative research, Reproductive Health Services

## Abstract

**Introduction:**

Social support can mitigate the impact of stress and stigma before or after an abortion. However, stigma anticipation can limit access to in-person support. Informal online spaces can offer opportunities to address unmet support needs including supplementing in-person support lacking within stigmatised contexts. While earlier studies have explored content of posts comprising personal accounts of abortion, little is known about the nuances of how and to what end online spaces are navigated.

**Methods:**

Semi-structured interviews were conducted remotely (online or by telephone) with 23 women living in Scotland (aged 20–54 years) recruited through social media and online advertisements. Reflexive thematic analysis was supported by NVivo12 software.

**Results:**

Key themes: obtaining support that was unavailable from in-person networks; preparation for abortion; reducing feelings of isolation. The majority of participants independently searched online for accounts of abortion, with only three receiving any signposting to specific resources. Without guidance, finding relevant, supportive content was not straightforward. The search process was additionally complicated by the prevalence of abortion stigma online, which generated an additional burden at a potentially challenging time. Those who received direction towards particular resources reported primarily positive online experiences.

**Conclusions:**

While online content could address perceived in-person support gaps, the process of finding supportive content without guidance can be complex. Online searching may also expose women to stigmatising material and interactions. Signposting by abortion services towards well-moderated and trustworthy online resources could be constructive in limiting exposure to stigma and misinformation, while allowing those seeking it to access better support.

**WHAT IS ALREADY KNOWN ABOUT THIS TOPIC**

- Online spaces present an opportunity to seek support from those outwith in-person social networks, for those seeking stigmatisable components of healthcare, including abortion.

**WHAT THIS STUDY ADDS**

- Finding relevant accounts of abortion was challenging, creating additional burden and confusion, exacerbated by a proliferation of anti-abortion rhetoric. Resource signposting ameliorated this difficulty.

**HOW THIS STUDY MIGHT AFFECT RESEARCH, PRACTICE OR POLICY**

- These findings illustrate the positive impact of guidance towards supportive abortion-related online content and advocate for healthcare professionals to signpost to online resources as standard.

## Introduction

Abortion is in high demand and is a safe procedure where legal and accessible.^1^ Yet, even in contexts where abortion has long been provided almost exclusively through state health services (as is the case in Scotland), abortion remains widely stigmatised and subject to unwarranted legal restrictions, presented across public discourse as contrary to norms of femininity.^2 3^ In addition to stigma perpetuated by societal institutions, those seeking abortion can face scrutiny and maltreatment through individual interactions with both network members and strangers alike.^4–6^ The stigmatisation of abortion – whether internalised, anticipated or enacted – limits the extent to which abortion experiences are widely shared.^7^ In concealing abortion, opportunities to seek social support (eg, validation, advice, etc.) and find others with similar experiences may be significantly restricted,^8^ often negatively impacting the abortion experience.^9 10^ In the absence of this in-person support, online spaces offer the opportunity to address these unmet support needs.

While formalised healthcare-based websites provide information about accessing abortion care and the procedure itself, informal online spaces for abortion-related content (such as forums or pages within a broader social media platform) can offer two key advantages over, or alongside, in-person interaction. First, they might provide an important alternative forum for communication and support, in which anonymity can be capitalised on, allowing platform users to share their story or learn from others without being identifiable.^11–13^ Users also can control the extent to which they engage with material, either browsing anonymously, responding through paralinguistic digital affordances (namely one-click actions such as ‘likes’) and comments, or creating their own post.^14^ Second, geographical and temporal boundaries of support-seeking are less relevant in virtual contexts, where content can be accessed at any time from anywhere.^11^ These affordances of online spaces have previously been explored as motivating factors in the decision to share stigmatisable health-related experiences online,^15–17^ but have not been directly applied to abortion.

Research into the ways in which abortion experiences are shared online has highlighted specific platforms (eg, Twitter, Reddit) as forums through which abortion can be normalised and support is offered.^18–21^ Using content analyses, these studies do not address the experiences and interpretations of users themselves. Our article addresses this gap by exploring women’s accounts of how and why they used online spaces to explore abortion experiences.

Specifically, in this article we address three research questions. (1) Why do women who have undergone abortion seek abortion-related content online? (2) How do they find and access that content? (3) Wwhat is their experience of online anti-abortion rhetoric?

## Methods

We devised a qualitative study to explore experiences of online social support-seeking around abortion. RVW-L conducted semi-structured interviews via telephone or Zoom (with or without video) at the participant’s preference. The University of Glasgow College of Social Science Ethics Committee in January 2020 (Ref. no.: 400190087) granted ethical approval of this study.

The recruitment criteria were purposefully broad to include a variety of abortion experiences. No time limit was specified on when participants had sought abortion, though participants were required to be aged 18+ years when interviewed. We recruited participants from across Scotland and shared recruitment information through social media and online advertisements. Prior to the interviews, RVW-L obtained consent through a digital consent form, with verbal consent obtained prior to commencing the interview.

RVW-L conducted 23 interviews between May and August 2020 (average duration 104 min, range 49–158 min). Informed by the literature, a flexible topic guide addressed general abortion experiences, and experiences of reading/interacting with accounts of abortion posted by others online. An iterative process of reflexive thematic analysis generated common themes.^22 23^ RVW-L (a PhD researcher with previous experience conducting abortion-related research) conducted the initial analysis, with thematic refinement and interpretation subsequently developed in collaboration with all the other authors.

### Patient and public involvement statement

Study development was informed by findings from the first author’s earlier research with women on their experiences of sharing abortion experiences with in-person networks (RV Wilson-Lowe, Women’s experiences of abortion disclosure: motivation, stigma, and social support, 2018, unpublished data). No patients were directly involved in the design, conduct or reporting of this study.

## Results

All participants identified as cisgender women, resided in Scotland at the time of the study, and had undergone at least one abortion (two participants reported more than one abortion). Sample characteristics are outlined in table 1. All reported browsing abortion-related online content posted by others, and 10 participants described sharing their own abortion experiences online, utilising a wide variety of informal online spaces such as blogs, forums and social media platforms. Only three participants described receiving signposting from health professionals or social network members towards online resources. Where data excerpts are presented, participants are referred to using a pseudonym and whether they reported signposting.

**Table 1:** Sample characteristics: demographics and online activity

Characteristic	n (N=23)
Age* (years)	
20–24	4
25–34	13
35+	6
Online activities	
Read content only	13
Shared and read	10
Rurality of residence†	
Rural (remote and accessible)	2
Small towns (remote and accessible)	1
Other urban areas	2
Large urban areas	18
Ethnicity	
White: Scottish	14
White: British	2
White: Other (including Gypsy and Traveller)	4
Asian, Asian Scottish or Asian British	2
Black: British, African or Caribbean	1
Scottish Index of Multiple Deprivation score‡	
First quintile (lowest)	9
Second quintile	6
Third quintile	1
Fourth quintile	3
Fifth quintile (highest)	4
Religious affiliation	
None/Atheist/Agnostic	18
Muslim	2
Christian	3
Sexual orientation	
Bisexual	5
Heterosexual	18
Formal education level (highest qualification received)	
High school	8
Trade/technical/vocational training	2
Undergraduate degree	9
Further degree(s)	4
Current employment status (multiple answers allowed)	
Employed for wages	17
Self-employed	2
Out of work/unable to work (at time of interview)	3
Student	3

*Participants' ages were in the range 20–54 years.

†Using the Scottish Government Urban Rural Classification 2020 (a '6-fold' classification that distinguishes between urban, rural, and remote areas with six categories).

‡The Scottish Index of Multiple Deprivation is an indication of socioeconomic status that measures across seven domains: current income, employment, health, education, skills and training, housing, geographic access and crime.

Through our analysis, we generated three key thematic areas regarding participants’ experiences of exploring online abortion-related content: (1) ‘Why go online: the appeal of personal accounts’, (2) ‘Accessing abortion accounts and the impact of signposting’ and (3) ‘Anti-abortion rhetoric and its impact’. There were notable differences in the experiences of those participants (3/23) who had been directed towards specific online spaces by healthcare professionals or friends – such as forums run by charitable organisations or closed Facebook groups – and those who had not. Hence, whether or not this direction had been received is used to categorise and compare experiences in the analysis presented below.

### Why go online: the appeal of personal accounts

Participants reported that they had explored online abortion accounts from the point at which they discovered their pregnancy to many years after their abortion, though this varied considerably across the sample. A desire to access social support from those with experiential knowledge of abortion was identified as a key driver towards online spaces, regardless of whether direction to specific online resources was reported. Support from in-person sources was limited by a perceived absence of those with direct experience of abortion within participants’ social networks. While some participants suggested that the taboo around abortion may have restricted their knowledge of who in their life had undergone abortion, isolation nevertheless resulted.

*“You kind of feel like you’re the only person in the world going through this. I’d never met anybody, or I had never knowingly met anybody at that stage. It felt like something very unusual […] I just felt an incredible sense of relief when I logged on and read so many other stories that had so many similarities to my own.”* [Nora, guidance received]

Without this knowledge, viewing online accounts was presented as a way to reduce feelings of loneliness without feeling obliged to make public that they had undergone, or were about to undergo an abortion, thereby avoiding potential stigmatising interaction.

*“Well, I didn’t want to tell my friends, because three quarters of them are Catholic […] and I didn’t want to be judged […] So I think it* [the decision to go online] *was more, it was all focused around the fact, I didn’t want people that were really close to me to know.”* [Hannah, no guidance]

*“I wanted real people with real situations and experiences […] rather than the kind of … I suppose the kind of … I don’t know, the official kind of advice or information that they give you kind of … I always felt like you would get more real answers from the people that have been through it.”* [Heather, no guidance]

### Accessing abortion accounts and the impact of signposting

To benefit from online accounts of abortion, participants first had to find this content. Two participants who had terminations for medical reasons (TFMR) reported being directed to a pregnancy-loss charity by their abortion providers. These spaces, with accounts that echoed their circumstances, were experienced positively. In Nora’s [guidance received] words, she “was met with unbelievable compassion and understanding” by those who had been through similar experiences with TFMR.

Another participant who did not undergo TFMR, but was directed to an online space by a friend, described a similarly positive experience.

*“Loads of women write on it* [closed Facebook group] *about abortion […] Like they talk about the nitty gritty detail like, some people even send pictures of like, oh this is a blood clot I had this morning […] It was refreshing to see people talk about stuff honestly, but also in a way where they were almost proud.”* [Alice, guidance received]

Without signposting, accessing support online was framed as a burdensome process, complicated by the vastness of the internet. Participants described a generally indiscriminate search strategy using language such as “stumbled on” [Anastasia, no guidance] and “trawling Google” [Melanie, no guidance] that expressed the haphazard nature of locating abortion accounts without direction. Broad search terms generated a large number of results requiring significant time and effort to sift through.

Variability in abortion provision internationally was described by participants as introducing another degree of difficulty in finding content relevant to the Scottish context. For example, after reading posts of users from the USA, Laurel described thinking that she would be able to go home after having misoprostol administered at a clinic. As her abortion took place prior to home use of abortion medications being introduced in Scotland this was not the case, which led to confusion and distress.

*“I found some quite conflicting advice, because I had found like American stories and things, and there was quite a lot of American stories that had mentioned that they were allowed to go home after the second tablet and just allow it to pass at home and I thought see if I could do that […] like I can just be at … in the house and in my own space and be more comfortable.”* [Laurel, no guidance]

#### Anti-abortion rhetoric and its impact

Exposure to anti-abortion rhetoric online appeared to be moderated by whether guidance toward relevant spaces was received, or if participants searched independently for support. For those who reported no guidance, finding relevant first-hand accounts of abortion was complicated by the prevalence of anti-abortion rhetoric. Without guidance toward supportive spaces, participants relied on broad search strategies for abortion more generally, which often returned explicitly anti-abortion content: “If you Googled ‘termination’ or ‘abortion’, I think probably the more […] negative and pro-life stuff comes up or came up at the time.” [Fiona, no guidance].

Anti-abortion rhetoric encountered online included harmful stereotypes and threats of violence, with some participants expressing that this negatively impacted their abortion experience and self-perception more broadly.

*“Then you hear ‘murderer’, you hear horrible derogatory terms towards women and threats and that sort of thing, they stand out and they’re really scary, and that’s kind of what warps your idea about all these things. […] Because you have seen all these horrible comments, you have convinced yourself that they’re right, that you are doing the worst thing in the world.”* [Rebecca, no guidance]

In contrast, the spaces participants were signposted to were framed as explicitly ‘safe’ space in which to explore others’ abortion experiences: “It’s a very safe space, very well moderated and you're not going to get in unless you've been through something like that…” [Delilah, guidance received]. Those without guidance recognised the potential value of clear initial guidance to sources which might incorporate trustworthy, experiential information recommended by healthcare professionals.

*“So I think it would be good to have somewhere where there was, like … even if it was just* [that] *the NHS kind of pointed you in the direction of a website that had these experiences, that didn't even necessarily be facilitated by the NHS. But I feel like, you know, you go on Google and you're going to get such a huge, wide range of random stuff and it’s like, we need signposting I think would be really helpful for people.”* [Fiona, no guidance]

## Discussion

To our knowledge, this is the first study to explore the nuance of experiences of seeking online social support around abortion. Women were motivated to use online resources to access experiential knowledge of abortion which, for them, constituted a form of support that was perceived as unavailable from their in-person networks. This emotional and cognitive support validated their experiences of abortion, demystifying and normalising abortion, and in doing so exploring abortion-related content online has the potential to play a destigmatising role in the abortion experience. The perceived benefits of using online spaces for support from others with similar experiences has been noted in previous studies, with specific consideration for stigmatisable healthcare experiences and identities.^16 17^ Those who had not received initial signposting described difficulties in finding supportive, ‘safe’ spaces containing relevant accounts, whereas participants who had received guidance generally reported positive experiences engaging with online content and a positive impact on feelings of isolation.

These findings highlight the potential role for abortion and other healthcare providers in facilitating supportive interactions online and limiting exposure to harmful content through signposting to vetted resources with personal accounts of abortion. Initial direction could assist women in finding informational and emotional support online before, during and after an abortion without the temporal or geographical limitations of in-person support.^11 24^ This could contribute towards a sense of continued support and social connectedness, which may positively impact abortion experiences, even if not directly supplied by health professionals.^25^

Recommending online resources represents a challenge for health services, in that the online space must be supportive and factual. Legitimate concerns may be held, for example, around signposting to services which purport to offer ‘support’, but which may in fact be anti-abortion ‘crisis pregnancy centres’. However, current National Health Service (NHS) practice provides signposting to online spaces in some instances. This was demonstrated by those in the sample who had TFMR, but also is practised by the NHS in regard to other stigmatisable healthcare experiences such as suicide.^26 27^ To support implementation of this practice nationally, vetting of potential online spaces would be necessary. Initial recommendations could be made to reputable existing evidence-based resources from organisations such as the British Pregnancy Advisory Service (BPAS), MSI Reproductive Choices or Abortion Talk. Initial guidance to trustworthy sources could help reduce the burden described in this study. Clinical guidelines for abortion care should encourage healthcare professionals to signpost service users to reliable online resources as standard practice.

A limitation of this study is the potential bias due to the remote recruitment and interview methods potentially excluding those without digital access (eg, in rural or deprived areas). Despite these concerns, the sample included substantial representation from these groups. Conversely, remote interviews could also be considered a strength of this study, as they were framed by some as enabling participation with no travel time or childcare, and allowing enhanced anonymity for those who chose audio-only methods.

## Conclusions

Personal abortion experiences shared online were identified by participants as a valuable source of support that may be perceived as unavailable from in-person sources. This study highlights the challenge faced in seeking supportive abortion-related content online, complicated by the wide and varied scope of online spaces and presence of anti-abortion rhetoric. Supportive experiences could be more readily accessible with signposting by healthcare professionals to previously appraised digital spaces. Criteria for assessment might include accurate and well-moderated content to encourage positive communication between users while limiting harassment.

## Data Availability

Data are available upon reasonable request.
